# Propofol Compared to Midazolam Sedation and to General Anesthesia for Percutaneous Microwave Ablation in Patients with Hepatic Malignancies: A Single-Center Comparative Analysis of Three Historical Cohorts

**DOI:** 10.1007/s00270-019-02273-y

**Published:** 2019-06-26

**Authors:** Robbert S. Puijk, Valentijn Ziedses des Plantes, Sanne Nieuwenhuizen, Alette H. Ruarus, Laurien G. P. H. Vroomen, Marcus C. de Jong, Bart Geboers, Caroline J. Hoedemaker-Boon, Deirdre H. Thöne-Passchier, Ceylan C. Gerçek, Jan J. J. de Vries, Petrousjka M. P. van den Tol, Hester J. Scheffer, Martijn R. Meijerink

**Affiliations:** 1 Department of Radiology and Nuclear Medicine, Amsterdam University Medical Centers - location VUmc, De Boelelaan 1117, 1081 HV Amsterdam, The Netherlands; 2 Department of Anesthesiology, Amsterdam University Medical Centers - location VUmc, De Boelelaan 1117, 1081 HV Amsterdam, The Netherlands; 3 Department of Surgical Oncology, Amsterdam University Medical Centers - location VUmc, De Boelelaan 1117, 1081 HV Amsterdam, The Netherlands

**Keywords:** Colorectal liver metastases (CRLM), Hepatocellular carcinoma (HCC), Microwave ablation (MWA), Propofol sedation, Moderate conscious midazolam sedation, General anesthesia, Local tumor progression (LTP), Local tumor progression-free survival (LTPFS)

## Abstract

**Purpose:**

In percutaneous ablation procedures, periprocedural pain, unrest and respiratory concerns can be detrimental to achieve a safe and efficacious ablation and impair treatment outcome. This study aimed to compare the association between anesthetic technique and local disease control in patients undergoing percutaneous microwave ablation (MWA) of colorectal liver metastases (CRLM) and hepatocellular carcinoma (HCC).

**Materials and Methods:**

This IRB-exempted single-center comparative, retrospective analysis of three cohorts analyzed 90 patients treated for hepatic malignancies from January 2013 until September 2018. The local tumor progression-free survival (LTPFS), safety and periprocedural pain perception were assessed using univariate and multivariate Cox proportional hazard regression analyses to correct for potential confounders.

**Results:**

In 114 procedures (22 general anesthesia; 32 midazolam; 60 propofol), 171 liver tumors (136 CRLM; 35 HCC) were treated with percutaneous MWA. Propofol and general anesthesia were superior to midazolam/fentanyl sedation regarding LTPFS (4/94 [4.3%] vs. 19/42 [45.2%] vs. 2/35 [5.7%]; *P* < 0.001, respectively). Local tumor progression rate was 14.6% (25/171). Eighteen tumors (72.0%) were retreated by ablation. Of them, 14 (78%) were previously treated with midazolam. Propofol versus midazolam (*P* < 0.001), general anesthesia versus midazolam (*P* = 0.016), direct postprocedural visual analog pain score above 5 (*P* = 0.050) and more than one tumor per procedure (*P* = 0.045) were predictors for LTPFS. Multivariate analysis revealed that propofol versus midazolam (HR 7.94 [95% CI 0.04–0.39; *P* < 0.001]) and general anesthesia versus midazolam (HR 6.33 [95% CI 0.04–0.69; *P* = 0.014]) were associated with LTPFS. Pain during and directly after treatment was significantly worse in patients who received midazolam sedation (*P* < 0.001).

**Conclusions:**

Compared to propofol and general anesthesia, midazolam/fentanyl sedation was associated with an increased periprocedural perception of pain and lower local tumor progression-free survival. To reduce the number of repeat procedures required to eradicate hepatic malignancies, general anesthesia and propofol sedation should be favored over midazolam.

## Introduction

The role of anesthetic techniques in percutaneous tumor ablation procedures is a highly debated topic worldwide since it may have impact on pain, anxiety and intraprocedural patient’s movements, thereby achieving an adequate, complete ablation zone (ideally a > 5 mm circumferential safety margin) [1–8] Several anesthetic methods can be used, such as general anesthesia, spinal anesthesia, and sedation using midazolam/fentanyl (hereafter: midazolam) or propofol ( ± intravenous analgesia) [1, 3]. The choice of anesthetic technique differs among institutes and is often based on the clinician’s and patient’s preferences and local availability. General and spinal anesthesia are invasive techniques which require specialized actions and are associated with higher systemic complication rates compared to sedation [1, 9]. Midazolam and propofol sedation are known for their short time to onset of action and short time to clearance [10, 11]. Moderate conscious sedation with midazolam was prospectively reported to be safe during biliary, tunneled catheter, diagnostic and vascular interventional procedures [12]. However, midazolam sedation tends to be associated with agitation, irregular breathing, respiratory depression and thoracic movement, which might lead to inadequate needle placement, needle tracking and creation of an insufficient tumor-free ablation margin [13, 14].

Over the past 15 years, propofol has become the drug of choice for many outpatient and short procedures, mainly due to its favorable pharmaceutical properties [3]. Guidelines for diagnostic and therapeutic purposes in gastrointestinal endoscopy were the first to describe clear consensus on sedation management with propofol [15–18]. Also in pediatric diagnostic imaging studies, compared to midazolam sedation, propofol is preferred in order to reduce undesired motion artifacts [19].

However, to our knowledge, there is no consensus which anesthetic technique should be used for an image-guided percutaneous liver ablation procedure, since there are no comparative studies evaluating the impact of anesthetic technique on local disease control and oncological outcomes. The aim of this study was to retrospectively analyze safety, efficacy and periprocedural perception of pain following percutaneous microwave ablation (MWA) for hepatocellular carcinomas (HCCs) or colorectal liver metastases (CRLMs), of the three most-used techniques in current-day clinical practice: general anesthesia, midazolam and propofol sedation.

## Materials and Methods

### Study Design and Population

This single-institution retrospective cohort study was conducted at Amsterdam University Medical Centers - location VUmc, the Netherlands, a tertiary referral center for hepatic malignancies.

**Fig. 1:**
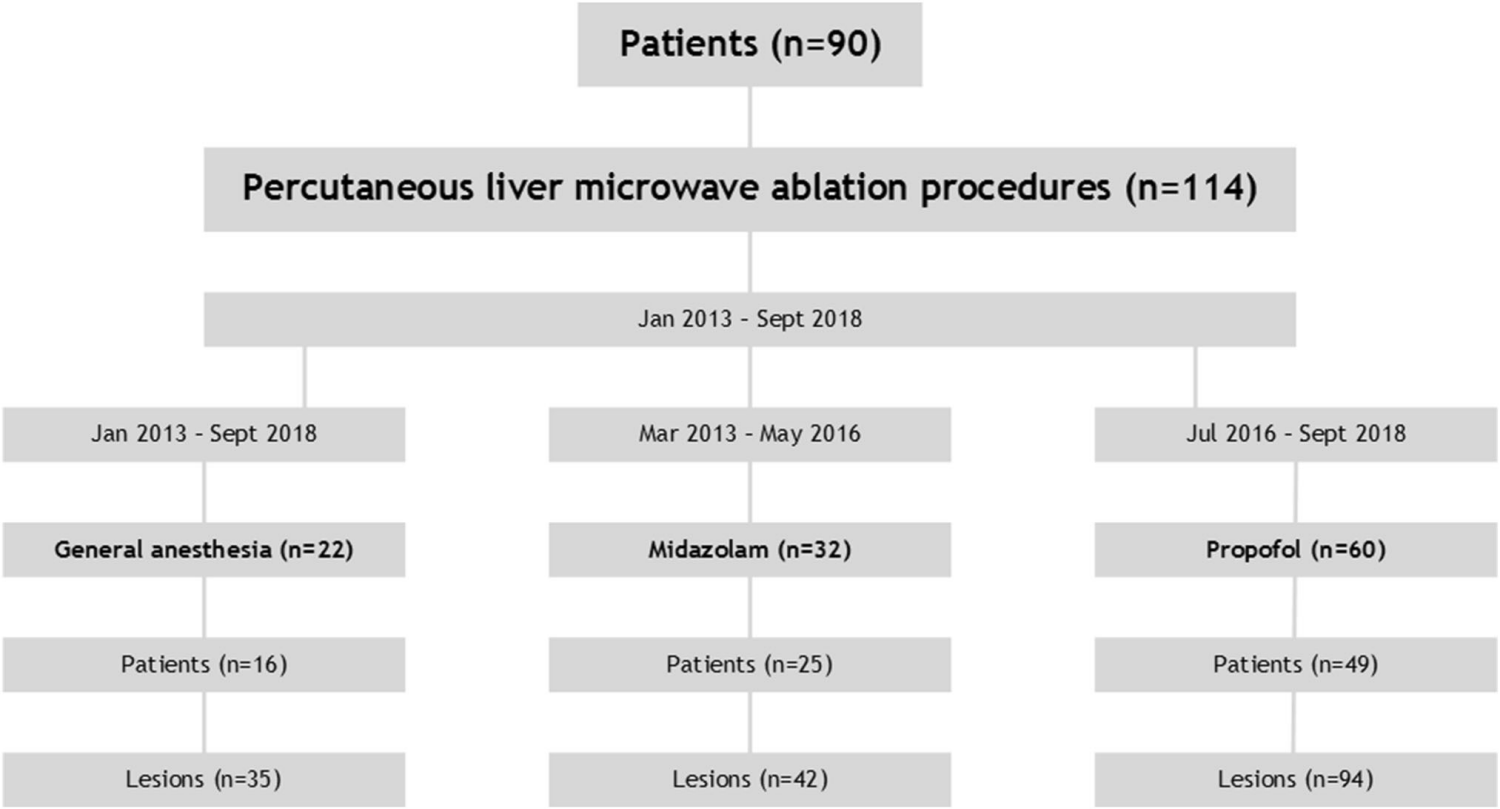
Flowchart for patient selection

Data were collected from a prospectively maintained liver tumor ablation registry. For reporting study data, the STROBE guidelines were followed [20]. Between January 2013 and September 2018, 90 consecutive patients (22 HCC; 68 CRLM) with 171 liver lesions underwent 114 percutaneous microwave ablations (Fig. 1). All patients were treated in our ambulatory interventional oncology suite, which houses a CT scanner and anesthetic facilities.

Patients were included if they were treated with computed tomography (CT)-guided percutaneous microwave ablation of either primary or secondary liver cancer. Clear documentation of anesthetic technique and medication doses were requirements for inclusion. Follow-up should have consisted of at least one imaging modality study to exclude local tumor progression (LTP). Patients treated with radiofrequency ablation were excluded, as this modality was being used less frequently over the last years as a result of clinicians’ preferences.

Although general anesthesia was mostly used for patients with contraindications for sedation, the choice for midazolam or propofol sedation was based on the availability of a specialized anesthetic assistant (propofol sedation) versus an interventional radiologist certified in administering midazolam sedation.

### General Anesthesia

Between January 2013 and September 2018, 22 procedures were performed with general anesthesia. Intravenous propofol (Diprivan^®^, AstraZeneca BV, Zoetermeer, the Netherlands), rocuronium (Esmeron^®^, Sandoz BV, Almere, the Netherlands), remifentanyl (Ultiva^®^, Mylan BV, Amstelveen, the Netherlands) and sufentanil (Sufenta^®^, Janssen Pharmaceutica, Beerse, Belgium) doses were commissioned by an anesthesiologist. General anesthesia included intubation and controlled respiration with continual cardiopulmonary monitoring. General anesthesia was chosen for patients with contraindications for both sedation techniques.

### Midazolam Sedation

Between March 2013 and May 2016, all procedures were performed with midazolam/fentanyl sedation (Fig. 1). Intravenous midazolam (Dormicum^®^, Roche BV, Woerden, the Netherlands) sedation doses were commissioned by the primary treating interventionist and administered by an anesthetic trained technician responsible for monitoring the patient’s vital functions. Starting dose of midazolam was 1–2.5 mg. Fentanyl (Durogesic^®^, Janssen Pharmaceutica, Beerse, Belgium) was given intravenously prior to the actual procedure (50 µg) and intraprocedurally when the patient was considered to experience pain (grimacing or body movements). Both doses were titrated and adjusted to body mass index (BMI) and clinical response. All patients received local anesthesia with an one-time bolus injection of lidocaine (B. Braun Medical B.V., Oss, the Netherlands). Respiratory depression was treated with active waking of patients, or when unsuccessful, temporary mechanical cuff breathing assistance. Flumazenil (Anexate^®^, Roche BV, Woerden, the Netherlands) and naloxone (Narcan^®^, Bristol-Myers Squibb BV, Utrecht, the Netherlands) were respectively available for potential midazolam and fentanyl overdosing.

When midazolam sedation was being performed in our institution, availability of anesthesiologists was insufficient. The first graduated group of certified anesthesia assistants (sedation specialist) following a dedicated training program to use target controlled infusion of propofol became available mid-2016.

### Propofol Sedation

From July 2016 until September 2018, propofol (Diprivan^®^, AstraZeneca BV, Zoetermeer, the Netherlands) was administered and monitored by a specialized anesthetic assistant using target controlled infusion, which automatically calculates the effective concentration of propofol in the patient’s cerebrum depending on the patient’s age and weight. Alfentanil (Rapifen^®^, Janssen Pharmaceutica, Beerse, Belgium) or remifentanil (Ultiva^®^, Mylan BV, Amstelveen, the Netherlands) was administered under the same circumstances as for midazolam sedation. Patients were allowed to breathe spontaneously, and the propofol infusion rate was titrated according to clinical response. Adequate sedation was considered to be reached by the absence of body movements and failure to respond to verbal commands. Although patients cannot comply to breathing instructions, propofol is known to create a tranquil, steady respiration status with minimal diaphragm movements despite the pain stimulus during probe placement dissimilar to midazolam sedation.

In case of inadequate sedation, additional propofol boluses were administered by increasing the carbon equivalent value. Some anesthesiologists preferred administration of additional esketamine (Ketanest-S^®^, Pfizer BV, Capelle aan den Ijssel, the Netherlands). All patients received local anesthesia with a one-time bolus injection of lidocaine (B. Braun Medical B.V., Oss, the Netherlands). During the procedure, one anesthesiologist was available on demand.

### Microwave Ablation Details

Prior to the procedure, patients who received general anesthesia or propofol sedation were routinely checked by an anesthesiologist. All patients were fasted for at least 6 h prior to the procedure. MWA (Emprint Thermosphere; by Medtronic, Minneapolis, Minnesota, USA) was used according to its CE mark.

Real-time CT fluoroscopy was used for needle guidance and targeting of lesion(s), surrounding structures and to assess the enlarging ablation zone. Intraprocedural assessment of contrast agent (Xenetix 300; by Guerbet SA, Villepinte, France) via an arterial catheter placement in the common hepatic artery was used to improve lesion conspicuity on real-time CT imaging (CT arterial portography; CT hepatic arteriography). Just prior to the ablation, patients were admitted to the angiography suite for the arterial catheter placement. The sheath was removed directly after the procedure by placing a hemostatic closure device at the common femoral artery. This technique has been previously described in detail by van Tilborg et al. [21] Track ablation was performed to prevent potential bleeding and tumor seeding along the needle track [1].

After the procedure, patients were directly admitted to the surgical ward in case of sedation with midazolam. After general anesthesia and propofol sedation, patients first went to the post-anesthesia care unit to monitor vital parameters before they were admitted to the surgical ward. Postprocedural analgesia protocol was identical for all three cohorts. All patients remained admitted at least one night.

All ablations were performed by two interventional radiologists (MRM and JJV) who both have a master degree in image-guided tumor ablation (having performed and/or supervised > 100 thermal ablation procedures).

### Follow-Up

All patients underwent contrast-enhanced (ce) CT immediately after ablation to assess technical success and complications. In case of an incomplete ablation, additional MWA was performed to treat the residual unablated tumor tissue. Follow-up consisted of [18F]-fluoro-2-deoxy-d-glucose (^18^F-FDG) positron emission tomography (PET)—CT scans 3 monthly during the first year and every 6 months thereafter, according to national guidelines [22] and the reporting criteria for image-guided tumor ablation [23].

### Data Collection and Analysis

Patient’s general (health) status, characteristics per lesion and characteristics per procedure were retrieved from the electronic patient database (Table 1). Total procedure time (from induction of sedation until needle removal), periprocedural pain perception, complications and local tumor progression and survival data are reported in Tables 2 and 3.

Intraprocedural pain was subjectively rated (present/absent) and reported by the anesthesiologist and/or interventional radiologist by signs of discomfort (e.g., [non-]verbal expression of agitation, grimacing, body movements). Postprocedural pain was measured by the nursing staff and documented as a written description or a pain perception score (visual analog scale; VAS) from 0 (no pain), 1–2 (mild pain), 3–5 (moderate pain) and 5–10 (severe pain), according to the adopted guidelines [23, 24]. The first pain perception score was noted directly after the procedure when patients were able to communicate. Within six hours afterwards, the second score was routinely noted. Separately, VAS scores of 5 and higher were analyzed since these scores are associated with severe pain [24]. If there was only a written description of postprocedural pain available, these data were first interpreted and translated into an interchangeable numeric score (VAS) by an independent researcher (VZP) and reviewed by a second author (RSP) to assess for interobserver variability.

A thermal ablation procedure was considered technically successful after having delivered the energy as planned and showing no residual enhancement around the ablation zone on immediately obtained ce-CT imaging [23]. Technical effectiveness was defined as complete ablation of the hepatic lesion as shown on first follow-up imaging after the ablation. LTP was defined as the “appearance of tumor foci at the edge of the ablation zone, after at least one contrast-enhanced follow-up study has documented adequate ablation and the absence of viable tissue in the target tumor and surrounding ablation margin” [23]. Local tumor progression-free survival (LTPFS) was calculated from the time of treatment to LTP per lesion treated, with death being censored.

### Statistical Analysis

Statistics are reported as number (with or without percentage; %), median (interquartile range, IQR) or mean (standard deviation, ± SD). Continuous measures were compared using the Kruskal–Wallis test (^§^). Non-continuous variables were compared using the Pearson *χ*^2^ test (^‡^).

Survival rates were estimated using the Kaplan–Meier method, with comparisons made using the log-rank test. The proportional hazards assumption was tested graphically in order to evaluate parallelism of the survival curves. Factors associated with LTPFS were analyzed using univariate and multivariate Cox proportional hazard regression models. Factors (e.g., tumor diameter) which are known having an association with LTPFS and factors with *P* ≤ 0.20 in univariate analysis were entered into the multivariate analysis model to simultaneously adjust for other potential predictors. Hazard ratios (HR) and 95 percent confidence intervals (95% CI) were calculated. The significance level for all parameters was set at *P* ≤ 0.05. Statistical analyses were performed in consultation with an independent, blinded epidemiologist (MCJ) using SPSS^®^ Version 22.0 (IBM^®^, Armonk, New York, USA) [25].

## Results

### Baseline Characteristics

Between January 2013 and September 2018, 90 patients (68 CRLM; 22 HCC) underwent 114 percutaneous MWA procedures for liver tumors that were not previously ablated. Forty-eight patients had a history of liver surgery for CRLM or HCCs distant from the ablation site. Of all procedures, 22 were performed under general anesthesia, 32 with midazolam/fentanyl and 60 with propofol sedation. The average number of ablated lesions per procedure was 1.50 ± 0.88 (range 1–5), and the average size of the largest diameter was 17.2 mm ± 10.6 (range 3–48 mm). Mediation dosages are listed in Table 1. There were no cases of medication overdosing where reversal of the administered medication was required. There were no significant *P* values found.

**Table 1:** Baseline characteristics

	Entire cohort	General anesthesia	Midazolam	Propofol	*P *value
Number of patients	90	16	25	49	
Patient characteristics					
Gender (M:F)	69: 21	11: 5	18: 7	40: 9	0.463^‡^
Mean age ± SD^†^ (years)	66.9 ± 11.0	69.4 ± 11.3	64.4 ± 12.3	67.4 ± 10.2	0.521^§^
Body mass index* (kg/m^2^)	25.9 (5.3)	26.9 (8.5)	26.6 (6.3)	25.3 (4.4)	0.094^§^
ASA physical status, ≥ 3	23	5	4	14	0.426^‡^
Primary tumor type					0.560^‡^
CRLM	68	13	17	38	
HCC	22	3	8	11	
Location colorectal cancer, right-sided	12 (17.6%)	4 (30.8%)	2 (11.8%)	6 (15.8%)	0.361^‡^
Characteristics per lesion					
Number of lesions	171	35	42	94	
Primary tumor type, no. of lesions					0.821^‡^
CRLM	136 (79.5%)	28 (80.0%)	32 (76.2%)	76 (80.9%)	
HCC	35 (20.5%)	7 (20.0%)	10 (23.8%)	18 (19.1%)	
Mean diameter ± SD^†^ (mm)	17.2 ± 10.6	18.1 ± 11.1	17.6 ± 11.8	16.6 ± 9.9	0.791^§^
Largest diameter (mm), > 30	21 (12.3%)	5 (14.3%)	11 (26.2%)	5 (5.3%)	0.921^‡^
Tumor-free margin size (mm), 0–5	26 (15.2%)	5 (14.3%)	12 (28.6%)	9 (9.6%)	0.423^‡^
Perivascular location	12 (7.0%)	5 (14.3%)	5 (11.9%)	2 (2.1%)	0.167^‡^
Characteristics per procedure					
Number of procedures	114	22	32	60	
Tumor number, > 1	37 (32.5%)	7 (31.8%)	8 (25.0%)	22 (36.7%)	0.397^§^
Synchronous CRLM	33 (28.9%)	5 (22.7%)	9 (28.1%)	19 (31.7%)	0.718^‡^
Catheter-guidance	98 (86.0%)	20 (90.9%)	26 (81.3%)	52 (86.7%)	0.589^‡^
General anesthesia					
Mean propofol dose (mg), ± SD		1160 ± 637			
Mean rocuronium dose (mg), ± SD		78 ± 46			
Mean remifentanil dose (µg), ± SD		2235 ± 1338 (*n* = 12)			
Mean sufentanil dose (µg), ± SD		20 ± 12 (*n* = 10)			
Midazolam sedation					
Mean midazolam dose (mg), ± SD			4.5 ± 2.1		
Mean fentanyl dose (µg), ± SD			205 ± 102		
Propofol sedation					
Mean propofol dose (mg), ± SD				706 ± 344	
Mean alfentanil dose (µg), ± SD				372 ± 197 (*n* = 54)	
Mean remifentanil dose (µg), ± SD				248 ± 84 (*n* = 6)	
Mean esketamine dose (mg), ± SD				18.6 ± 10.3	

Values are reported as number (with or without percentage; %) or dose

*ASA* American Society of Anesthesiologists score, *CRLM* colorectal liver metastases, *F* female, *HCC* hepatocellular carcinoma, *kg* kilogram, *M* male, *mg* milligram, *mm* millimeter, *µg* microgram, *min* minutes, *VAS* visual analog scale, *y* year

^*^median (interquartile range, IQR) or ^†^mean (standard deviation, ± SD)

^‡^Pearson *χ*^2^ test between groups; ^§^Kruskal–Wallis test

Median follow-up time after each procedure for the general anesthesia group was 8.4 months (IQR 17.6), 23.3 months (IQR 26.8) for the midazolam group and 6.5 months (IQR 6.6) for the propofol group (Table 3). Of all 90 patients, 12 (13.3%) deceased during follow-up (general anesthesia, *n* = 6; midazolam, *n* = 5; propofol, *n* = 1). All patients died from progression of disease. In case of death, median time from last ablative therapy to death was 15.8 months (IQR 29.2).

### Complications

There were slightly more complications reported in the propofol group compared to the midazolam group (4 vs. 1, respectively; [*P* = 0.392]; Table 2). In both groups, one minor iatrogenic pneumothorax occurred due to the ablation devices. Those resolved spontaneously. Two procedures with propofol sedation were complicated by hepatic hemorrhages along the needle track. These patients were admitted for an emergency coiling procedure. In both cases, there was no lack of breathing control reported. The last complication, respiratory insufficiency, occurred due to postprocedural aspiration which required emergency intubation and recovery at the intensive care unit.

**Table 2:** Outcomes of all percutaneous liver tumor microwave ablation procedures

	Entire cohort	General anesthesia	Midazolam group	Propofol group	*P *value
Procedures	114	22	32	60	
Mean procedure time (min), ± SD	101 ± 50	108 ± 69	105 ± 63	97 ± 36	0.956^§^
Intraprocedural pain	12	–	11	1	< 0.001^‡^
First measured postprocedural pain (VAS)*	1 (0–8)	0 (0–5)	3 (0–8)	1 (0–5)	< 0.001^§^
Second measured postprocedural pain (VAS)*	1 (0–7)	0 (0–2)	2 (0–7)	0 (0–5)	< 0.001^§^
No. of procedures after which the *first* measured postprocedural pain (VAS) score was ≥ 5–10	12	1	10	1	< 0.001^‡^
No. of procedures after which the *second* measured postprocedural pain (VAS) score was ≥ 5–10	4	–	3	1	0.101^‡^
Intraprocedural complication(s)	5	–	1	4	0.392^‡^
Pneumothorax	–	–	1	1	
Bleeding	–	–	–	2	
Respiratory insufficiency	–	–	–	1	

Statistics are reported as number (with or without percentage; %)

*Min* minutes, *VAS* visual analog scale

^*^Median (interquartile range, IQR) or ^†^mean (standard deviation, ± SD)

^‡^Pearson *χ*^2^ test between groups; ^§^Kruskal–Wallis test

### Pain Perception

Intraprocedural pain occurred significantly more often in the midazolam group (11 out of 32 procedures, [34.4%]) compared to the general anesthesia (0%) and propofol groups (1.7%) (Table 2; [*P* < 0.001]). Pain scores after the procedures were significantly higher in the midazolam group (*P* < 0.001).

### Local Disease Control, Local Tumor Progression and Local Tumor Progression-Free Survival

Technical success was achieved in all 171 hepatic lesions (primary technique effectiveness of 100%), showing no residual enhancement around the ablation zone on immediately assessed ce-CT imaging.

Twenty-five out of 171 hepatic lesions (entire cohort 14.6%; general anesthesia, *n* = 2 [5.7%]; midazolam, *n* = 19 [45.2%]; propofol, *n* = 4 [4.3%]) showed LTP on follow-up imaging. Eighteen lesions (18/25 [72.0%]) in 10 patients were retreated by ablation (general anesthesia, *n* = 1 [5.6%]; midazolam, *n* = 14 [77.8%]; propofol, *n* = 3 [16.7%]). In six patients (7/25 [28.0%] locally progressed tumors), local reintervention was considered biologically futile because of concomitant distant progression (Table 3). For CRLM versus HCC, LTP was respectively detected in 22 out of 136 lesions (16.2%) versus 3 out of 35 lesions (8.6%) (*P* = 0.420). In case of local tumor progression, the mean time to detection of LTP was 5.7 ± 4.3 months (general anesthesia), 6.1 ± 4.8 months (midazolam) and 3.6 ± 0.7 months (propofol) (*P* = 0.230).

**Table 3:** Outcomes of all treated liver lesions

	Entire cohort	General anesthesia group	Midazolam group	Propofol group	*P *value
Number of patients	90	16	25	49	
Number of lesions	171	35	42	94	
Number of procedures	114	22	32	60	
Median follow-up after each procedure (months)*	8.9 (14.1)	8.4 (17.6)	23.3 (26.8)	6.5 (6.6)	< 0.001^§^
Local tumor progression (LTP; no. of lesions)	25 (14.6%)	2 (5.7%)	19 (45.2%)	4 (4.3%)	< 0.001^‡^
Time-to-local tumor progression					
Median for all lesions (months; 95% CI)*	Not reached	Not reached	Not reached	Not reached	NA
Mean time to detection of LTP^†^	5.6 ± 4.3	5.7 ± 3.1	6.1 ± 4.8	3.6 ± 0.7	0.230^§^
Repeat sessions (no. of re-ablated lesions)	18 (72.0%)	1 (5.6%)	14 (77.8%)	3 (16.7%)	0.769^‡^

Statistics are reported as number (with or without percentage; %)

*NA* not applicable

^*^median (interquartile range, IQR) or ^†^mean (standard deviation, ± SD)

^‡^Pearson *χ*^2^ test between groups; ^§^Kruskal–Wallis test

**Fig. 2:**
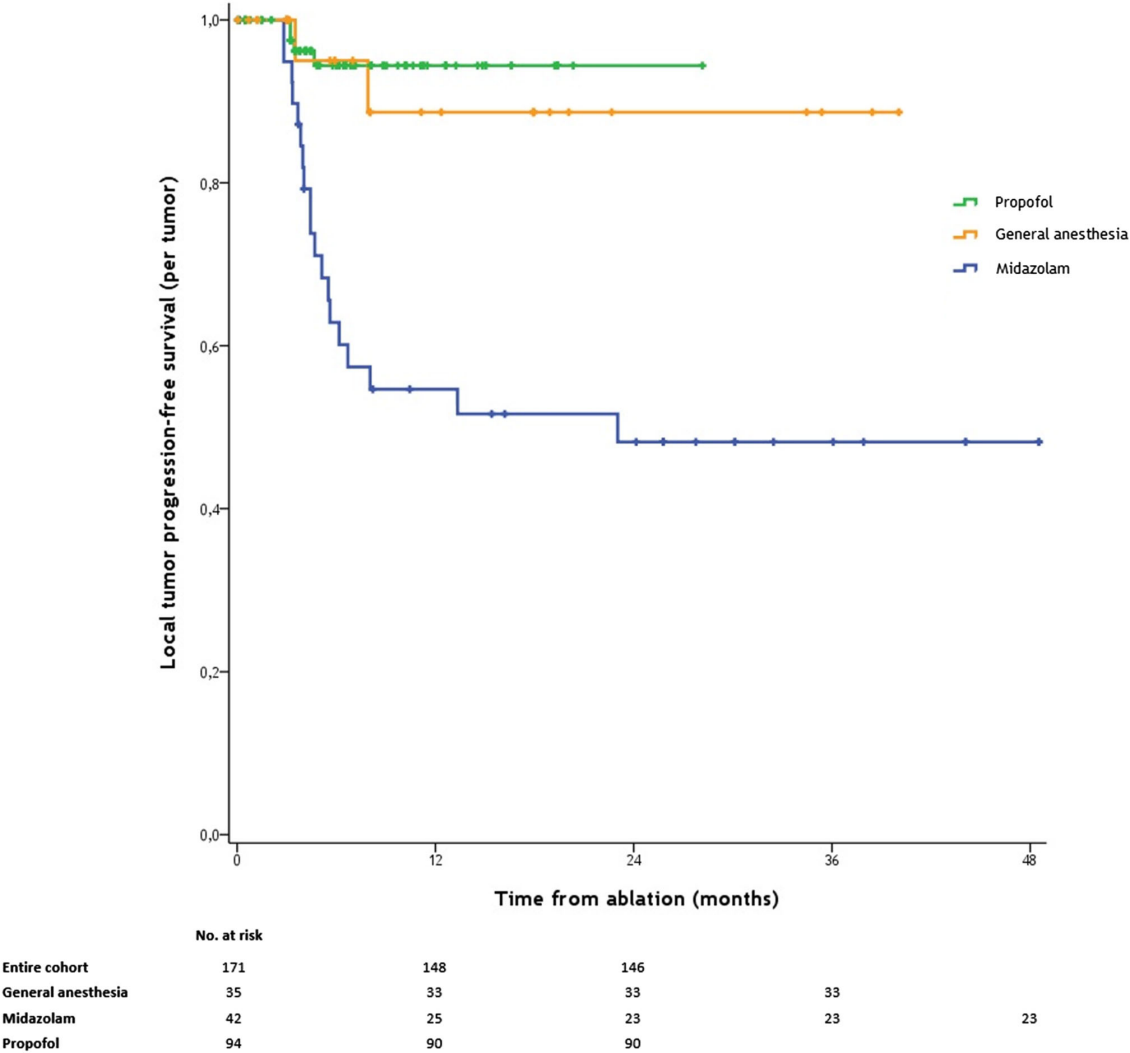
Kaplan–Meier curves indicating the survival time without local tumor progression (local tumor progression-free survival) per MWA-treated tumor. Kaplan–Meier curves showing freedom from local tumor progression (per-lesion) for patients with hepatic malignancies treated by percutaneous microwave ablation with either propofol sedation (green line), general anesthesia (orange line) or moderate conscious sedation with midazolam (purple line). Numbers at risk are MWA-treated tumors. Overall comparison log-rank (Mantel–Cox) *P* < 0.001. Death without local  tumor progression is censored

**Fig. 3:**
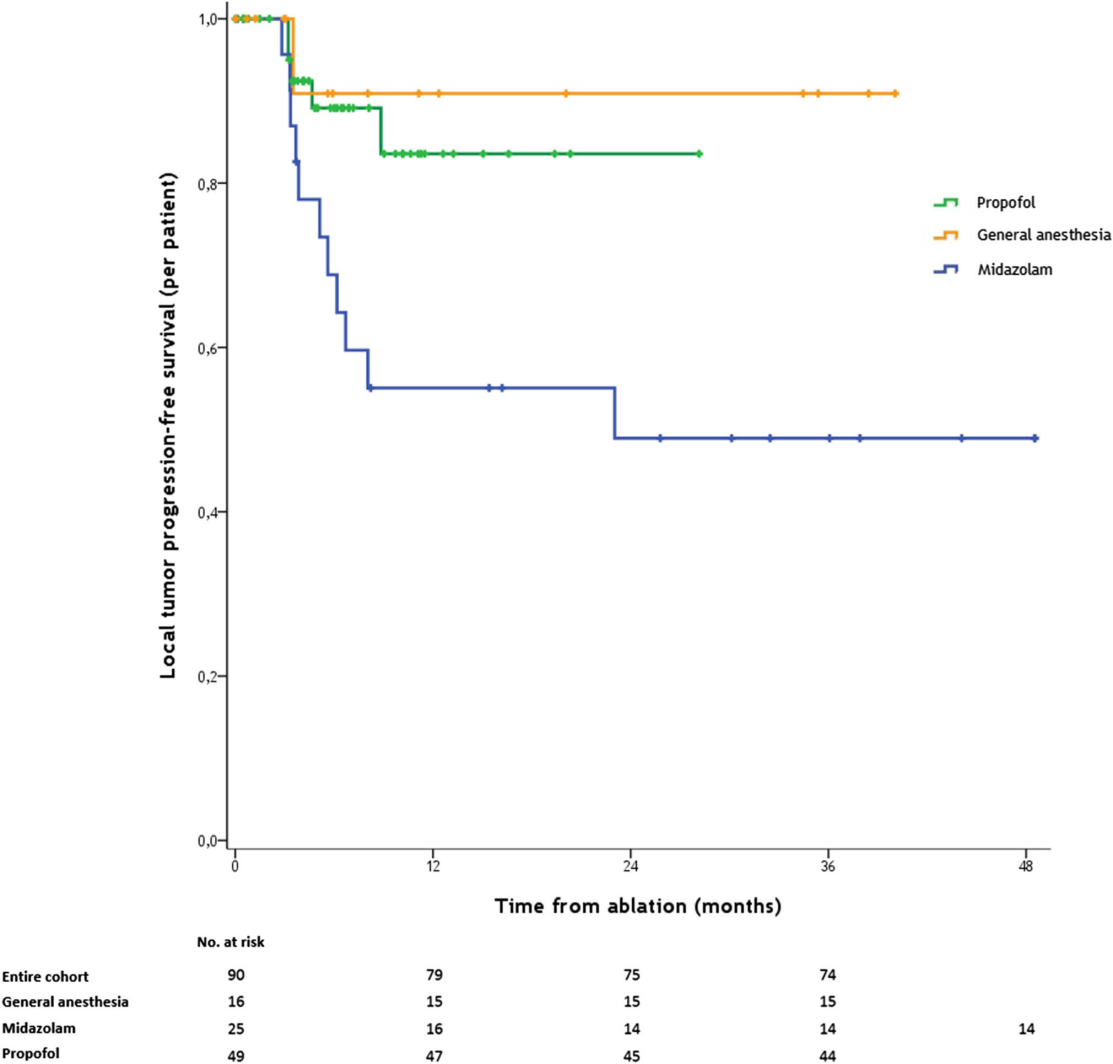
Kaplan–Meier curves indicating the survival time without local tumor progression (local tumor progression-free survival) per patient. Kaplan–Meier curves showing freedom from local tumor progression (per-patient) for patients with hepatic malignancies treated by percutaneous microwave ablation with either propofol sedation (green line), general anesthesia (orange line) or moderate conscious sedation with midazolam (purple line). Numbers at risk are patients. Overall comparison log-rank (Mantel–Cox) *P* < 0.019. Death without local tumor progression is censored

LTPFS (analyzed per tumor) significantly differed between the three groups (*P* < 0.001) (Fig. 2). Univariate and multivariate associations with LTPFS are shown in Table 4. For LTPFS, the HR after multivariate analysis was 7.94 (95% CI 0.04–0.39; [*P* < 0.001]) in favor of propofol versus midazolam sedation and 6.33 (95% CI 0.04–0.69; [*P* = 0.014]) in favor of general anesthesia versus midazolam sedation. Per-patient LTPFS results significantly differed between the cohorts (*P* < 0.019) (Fig. 3).

**Table 4:** Factors associated with local tumor progression-free survival identified by univariate and multivariate Cox regression analyses from the time of the ablation to local tumor progression

	Univariate analysis			Multivariate analysis		
	Hazard ratio	95% CI	*P* value	Hazard ratio	95% CI	*P* value
Patient characteristics						
Age	1.00	0.97–1.03	0.979			
BMI	1.01	0.94–1.09	0.743			
ASA, ≥ 3	0.96	0.40–2.30	0.928			
Primary tumor type	2.08	0.62–6.95	0.237			
Location colorectal cancer, right-sided	0.50	0.12–2.16	0.356			
Characteristics per lesion						
Mean diameter (mm)	1.02	0.99–1.06	0.266			
Largest diameter (mm), > 30	1.19	0.41–3.47	0.753			
Tumor-free margin size (mm), 0–5	1.23	0.46–3.29	0.679			
Perivascular location	1.86	0.56–6.23	0.313			
Characteristics per procedure						
Tumor number, > 1	2.45	1.02–5.87	0.045	2.03	0.20–1.19	0.117
Catheter-guidance	0.59	0.22–1.58	0.293			
Outcomes						
Intraprocedural pain	1.85	0.65–5.26	0.246			
Intraprocedural complications (other)	1.35	0.18–10.06	0.771			
First measured postprocedural pain, VAS ≥ 5–10	2.52	0.99–6.35	0.050	1.24	0.34–2.32	0.809
Anesthetic technique						
Propofol versus midazolam	8.70	0.04–0.34	< 0.001	7.94	0.04–0.39	< 0.001
General anesthesia versus midazolam sedation	5.99	0.04–0.72	0.016	6.33	0.04–0.69	0.014

Death without local tumor progression is censored

*ASA* American Society of Anesthesiologists score, *BMI* body mass index, *CRLM* colorectal liver metastases, *mm* millimeter, *VAS* visual analog scale

Variables with a *P *value ≤ 0.20 were entered in the multivariate analysis

## Discussion

Due to the expanding role of interventional radiology in liver cancer treatment, the amount and complexity of thermal ablation procedures have raised the demand for safe anesthetic management in the ambulatory interventional oncology suite [3]. This comparative analysis of three historical cohorts described the outcomes of patients undergoing percutaneous liver tumor MWA for CRLM or HCC to identify potential differences between general anesthesia, midazolam and propofol sedation.

Anesthetic technique was the most significant predictor of LTPFS in the Cox regression model in favor of propofol versus midazolam sedation (HR 7.94; *P* < 0.001) and in favor of general anesthesia versus midazolam sedation (HR 6.33; *P* = 0.014). This result suggests that patients who underwent a percutaneous procedure under general anesthesia or propofol sedation had an equally reduced risk of developing LTP compared to patients treated under midazolam sedation. These outcomes imply that propofol sedation results in fewer patient movements, better control of breathing and less pain compared to midazolam sedation. General anesthesia and deep sedation with propofol apparently lead to more controlled ablative procedures with superior precision in needle placement and needle tracking, presumably creating wider and more accurate ablation zones. General anesthesia is the ideal technique due to the fact that one can request apnea at any time with completely controllable respiration. Propofol appears to be equivalent regarding local control, although it is theoretically possible that the continued respiration during probe placement contributed to the two cases of probe-induced hepatic hemorrhage.

The efficacy following *percutaneous* MWA under propofol sedation and general anesthesia in this series is comparable to the per-lesion LTPFS reported in the most recent surgical series following *open* MWA for similar sized liver tumors [26]. This may indicate equipoise has been reached between the open and percutaneous approach.

Although the CIRSE quality improvement guidelines mention that thermal liver ablation can be performed under intravenous sedation and general anesthesia, to the best of our knowledge, this is the first study that has compared anesthesia techniques for liver tumor ablation [1]. Kim et al. retrospectively compared general anesthesia to midazolam/fentanyl sedation in a small number of renal cell carcinoma patients treated with percutaneous radiofrequency ablation [27]. The authors also reported a significantly higher LTP rate in the midazolam group, mainly caused by insufficient pain control and breath holding during the procedure leading to incomplete ablations.

Midazolam sedation is traditionally being used for interventional procedures because of its reported safety [12]. From a pharmacodynamics point of view, midazolam differs widely from propofol, which is known to achieve a more profound sedation level and shorter recovery time [28]. Several series compared midazolam sedation to propofol in interventional procedures. One outdated trial included 40 patients with intracranial vascular disease and randomized between the two [29]. No differences were found with regard to complications (pain, inappropriate movements and respiratory changes) and both patient’s and physician’s satisfaction score. However, another randomized study concluded that propofol sedation was associated with superior physician satisfaction (*P* < 0.05) and less respiratory depression and anxiety compared to midazolam for equivalent sedation levels in patients undergoing a percutaneous transluminal angioplasty (*P* < 0.05) [14].

In other medical fields, propofol is being used extensively for various procedures. In gastro-intestinal endoscopy, one meta-analysis of 22 studies reported that propofol sedation was associated with shorter recovery and discharge time and that patients were more likely to cooperate compared to traditional sedative agents [28]. One recently published, double-blind, randomized trial revealed that significantly fewer patients who received propofol remembered being awake during outpatient colonoscopy compared to midazolam sedation (respectively 2% vs. 17%, *P* < 0.0001) [30]. More patients who received propofol were “very satisfied” with their level of consciousness compared to midazolam (86.3% vs. 74%, *P* = 0.0005). Twenty-six percent of midazolam procedures were rated as “difficult” by the treating physician compared to 4.3% for propofol (*P* < 0.0001). Anesthesia-related complications were fewer in the propofol group (2.7% vs. 11.7%, *P* < 0.0001). Another randomized trial also reported less pain perception (*P* < 0.001) and greater patient and endoscopist satisfaction during colonoscopy in case of propofol-based sedation (*n* = 126) compared with midazolam/fentanyl (*n* = 136) [31].

Interestingly, several in vitro studies describe another potential advantage of propofol—that it may contribute to immune modulation, anti-inflammation and inhibition of cancer cell proliferation and invasion [32, 33].

This study has several limitations. First, the three groups are retrospectively analyzed; in other words, the anesthetic technique was not randomly allocated. As such, the possibility of selection bias is not negligible. Though all procedures were analyzed consecutively from a prospective registry database and even though univariate and multivariate analysis was performed to correct for potential biases, there are no guarantees that exclude residual confounding. Because general anesthesia was often chosen for patients with contraindications for both sedation techniques, assessing patient-based oncological endpoints such as overall or cancer-specific survival was considered untrustworthy. Intraprocedural pain perception contains subjective measurements which may have introduced recall bias. Whenever possible, data were reviewed separately by two researchers (RSP and VZP). Since periprocedural parameters, such as pain, were digitally reported by the anesthesiologist and nurse anesthetist, these factors were presumably being more extensively documented in the general anesthesia and propofol groups. In addition, monitoring and administration of midazolam/fentanyl were performed by the interventional radiologist, who, even though specifically trained and certified for this procedure, had limited knowledge of the systemic effects, while general anesthesia and propofol sedation were always administered by a specialized anesthetic assistant under direct supervision of an anesthesiologist.

Despite the fact that propofol administration should be reserved for anesthesia providers, a recently published survey showed that anesthesia providers are not uniformly available during interventional procedures [3]. This could result in situations where interventional radiologists are increasingly being involved in administering sedative drugs and managing complications, as was the case in our institution. Another limitation was the unequal median follow-up duration between the groups (*P* < 0.001); however, since the majority of LTPs appeared within the first 6 months post-treatment (Fig. 2, numbers at risk), the likelihood of developing LTP decreases over time (plateau curve). Although overall survival is generally considered the most relevant oncological endpoint, the efficacy of closely related ablation techniques to eradicate tumors can best be elucidated by comparing the time to LTP. Although multiple lesions in one patient cannot be considered independent, the per-patient analysis (counting LTP of one of the ablated lesions in a single patient as an event) showed equal differences between the three groups.

To conclude, propofol sedation represents a valid alternative to general anesthesia for percutaneous liver tumor ablation, and midazolam sedation does not. Midazolam sedation was inferior to both general anesthesia and to propofol with regard to local tumor control. Compared to midazolam sedation, propofol reduced the periprocedural perception of anxiety and pain, decreased patient movements and resulted in better control of breathing. This probably contributed to more precise needle placements and tracking with higher ablation accuracy, which is reflected by the superior LTPFS. Propofol-based sedation reduces the number of repeat procedures and should be favored over midazolam sedation in percutaneous liver tumor ablation. Future research should focus on the added value of innovative techniques such as one lung and high-frequency jet ventilation.

## Abbreviations

^18^F-FDG: [18F]-fluoro-2-deoxy-d-glucose
ASA: American Society of Anesthesiologists
BMI: Body mass index
CE: Contrast enhanced
CI: Confidence interval
CT: Computed tomography
CRLM: Colorectal liver metastasis
HCC: Hepatocellular carcinoma
HR: Hazard ratio
IQR: Interquartile range
LTP: Local tumor progression
LTPFS: Local tumor progression-free survival
MWA: Microwave ablation
PET: Positron emission tomography
SD: Standard deviation
VAS: Visual analog scale
